# Pseudophakic macular edema in nondiabetic and diabetic patients without diabetic retinopathy treated with intravitreal dexamethasone implant

**DOI:** 10.1186/s40942-023-00489-2

**Published:** 2023-09-18

**Authors:** Magna Vanessa Rodrigues, Jose Mauricio Botto Garcia, Katia Delalibera Pacheco, Fabricio Tadeu Borges, David Leonardo Cruvinel Isaac, Marcos Avila

**Affiliations:** 1 Centro Brasileiro da Visao, Brasilia, DF Brazil; 2 Federal University of Goias, Centro de Referencia em Oftalmologia (CEROF), Goiania, GO Brazil; 3 Centro Brasileiro de Cirurgia de Olhos, Goiania, GO Brazil

**Keywords:** Dexamethasone, Intravitreal injections, Macular edema, Cataract, Phacoemulsification

## Abstract

**Background:**

The purpose of this study was to compare the impact of intravitreal dexamethasone (DEX) implant during a 12-month period in nondiabetic and diabetic patients without diabetic retinopathy (DR) as a treatment for refractory pseudophakic cystoid macular edema (PCME) following prior treatment with topical nepafenac 0.1% and prednisolone 1%.

**Methods:**

Forty-two consecutive medical records of patients diagnosed with PCME after uneventful cataract surgery were included. The outcomes measured included best corrected visual acuity (BCVA) and central foveal thickness (CFT). Linear regression analysis was statistically applied.

**Results:**

Following topical treatment, nondiabetic and diabetic subjects presented a mean ± SD gain of − 0.11 ± 0.11 and − 0.18 ± 0.11 BCVA logMAR and a CFT reduction of − 43.42 ± 53.66 µm and − 58.76 ± 36.28 µm, respectively. The mean BCVA gain at month 12 subsequent to DEX implantation was − 0.35 ± 0.17 in nondiabetic (p < 0.001) and − 0.55 ± 0.26 in diabetic patients (p < 0.001), with CFT reductions of − 195.71 ± 93.23 µm (p < 0.001) and − 260.81 ± 198.69 µm (p < 0.001), respectively. Patients who responded with better VA after topical treatment presented better visual outcomes at month 12 following DEX implantation (r^2^ = 0.46; *rho* = − 0.71, p < 0.01).

**Conclusion:**

Nondiabetic and diabetic patients without DR demonstrated similar results after DEX implant after combined topical therapy, suggesting that selected diabetic patients may have a response comparable to that of nondiabetic patients with PCME.

## Background

Pseudophakic cystoid macular edema (PCME) is among the most common and feared complications after cataract surgery, even in the absence of intraoperative complications or other risk factors. Its incidence is about 1% among patients with no known risk factors [1–3]. Studies have identified diabetes, uveitis, epiretinal membrane, perioperative complications (posterior capsular rupture) as high-risk factors related to a higher incidence of PCME [4, 6].

Diabetes has traditionally been presented as a possible risk factor for the increased incidence of PCME after routine cataract surgery [7–10]. However, it has been suggested that diabetes itself might not impair recovery from uneventful surgery and may not increase the relative risk of PCME when compared to the risk in nondiabetic controls [2–8].

Fluorescein angiography (FA) and spectral domain optical coherence tomography (SD-OCT) represent the techniques of choice for evaluation and follow-up of macular status in both nondiabetic and diabetic patients [2]. In particular, SD-OCT offers both quantitative and qualitative information in a non-invasive and repeatable way, providing the central foveal thickness (CFT), widely used both in randomized clinical trials and in clinical practice [5–9].

Prophylactic regimens have been broadly studied to prevent PCME with variable results [11–15]. In diabetic subjects, topical combined therapy with nonsteroidal anti-inflammatory drugs (NSAIDs) and steroids seems to be superior to steroid monotherapy [7, 16]. Intravitreal dexamethasone (DEX) implant (0.7 mg, Ozurdex; Allergan, Inc., Irvine, CA) is a novel therapeutic strategy for PCME, especially in refractory patients who previously underwent usual therapies [12, 17–29]. However, there is a lack of data about DEX implants over long follow-up periods [12, 17, 19, 26, 27, 30].

The purpose of this study was to compare the impact of DEX implant during a 12-month period in nondiabetic and diabetic patients without diabetic retinopathy (DR) as a treatment for refractory PCME following prior treatment with topical combined therapy.

## Materials and methods

### Study design

A retrospective study included medical records of consecutive patients diagnosed with PCME after uneventful phacoemulsification with posterior chamber intraocular lens (IOL) implantation in two affiliated ophthalmic hospitals. This study adhered to the tenets of the Declaration of Helsinki and was approved by the institutional review board of the institutions involved. All patients have read and signed an informed consent form after receiving a detailed review of the benefits and complications of this off-label therapy, prior to each treatment.

A total of 58 eyes of 58 patients were initially identified as patients with PCME treated with dexamethasone implant from January 2016 to December 2018 according to the National Guidelines for the Management of Cataracts. To avoid statistical bias, only one eye (the first eye implanted with DEX) of each patient was included in the analysis, regardless the presence of bilateral PCME, present in 2 individuals. Data from 42 pseudophakic subjects with uneventful cataract surgery that still had PCME diagnosed, after at least 2 months follow-up period of topical treatment were included. Fourteen patients’ data were not included in the analysis due to exclusion criteria.

None of the patients had used NSAIDs or topical steroids prior to surgery, but all subjects were treated with topical nepafenac 0.1% and topical prednisolone 1% three times daily (tid) in the immediate postoperative period and for 2 months after diagnosis from PCME. Failure of topical treatment if CFT is 10% or more above baseline on SD-OCT (Spectralis, Heidelberg Engineering, Heidelberg, Germany) after 2 months of this treatment. The use of these eyedrops was discontinued after DEX implantation.

Nondiabetic patients (n = 21 eyes) were compared to those with diabetes without diabetic retinopathy (n = 21 eyes). Diabetic patients belonged to our regular screening system for diabetic retinopathy according to the International Council of Ophthalmology guidelines for diabetic eye care (updated in 2013).

The primary endpoint was the outcome of DEX implantation as a treatment for PCME in nondiabetic and selected diabetic patients, with the intent to establish the impact of diabetes on visual recovery and OCT improvements during a 12-month period after a 2-month course of topical combined therapy.

### Clinical data

Preoperative and postoperative best corrected visual acuity (BCVA) and intraocular pressure (IOP) data were recorded. BCVA was measured using Snellen charts and later converted into logMAR for statistical analyses. FA was performed to diagnose PCME prior to treatment with NSAIDs, after 2 months of topical treatment, prior to DEX implantation, and then after 6 months of the intravitreal implantation, as an adjuvant supporting the PCME diagnosis and the need for a second implant.

Only the medical records of patients who underwent SD-OCT after cataract surgery, after using topical therapy for 2 months and after implantation of DEX for a 12-month follow-up, were included.

The criteria used for diagnosis were any of the following after uneventful cataract surgery: the CFT was defined as the mean thickness in the central 1 mm diameter area, and PCME was defined as an increase in the CFT of 10% or more over the baseline value at any postoperative time point, with cystic changes or intraretinal fluid confirmed by SD-OCT for a minimum of 2 months, and FA depicting perifoveal petaloid staining with late leakage from the optic nerve head 2 months after cataract surgery.

Both groups of nondiabetic patients (n = 21 eyes) and diabetic patients without diabetic retinopathy (n = 21 eyes) received DEX implant (0.7 mg) in the studied eye and in case of retreatment. Retreatment with a second DEX implant was applied provided BCVA loss of more than one line was documented compared to the level measured after DEX implantation and if the increase in CFT was 10% or more above baseline, with cystic changes or intraretinal fluid confirmed by SD-OCT.

### Surgical data

Standardized phacoemulsification technique was used in all cataract surgeries. Each surgical procedure consisted of conventional phacoemulsification (Centurion®; Alcon, Fort Worth, Texas, USA), including a clear or near clear cornea incision, capsulorhexis, phacoemulsification followed by intraocular lens (single-piece foldable acrylic intraocular lens) placement in the capsular bag under monitored anaesthesia care with topical anaesthesia or retrobulbar anesthesia.

DEX implant (0.7 mg, Ozurdex; Allergan, Inc., Irvine, CA) is a sustained-release drug delivery system. The implant was injected under sterile conditions following povidone iodine 5% instillation using a sterile eyelid speculum and topical anaesthesia, in accordance with the manufacturer’s instructions.

### Inclusion criteria

The subjects included were those aged 40–80 years who were eligible for cataract surgery according to the National Guidelines for the Management of Cataracts (updated in 2003).

### Exclusion criteria

Diabetic patients with any form of diabetic retinopathy or diabetic nephropathy at any level described in medical record were excluded. Patients with prior or active wet age-related macular degeneration, retinal vein and/or artery occlusion, macular ischaemia, macular scarring related to subretinal fibrosis, corneal scarring, uveitis, and previous vitreous surgery were excluded.

Retinal detachment, retinal necrosis, vitritis and/or endophthalmitis, vitreous haemorrhage, retinal phlebitis, optic neuritis, previous intraocular surgery or procedures (including fundus laser photocoagulation), prior or scheduled antagonists of vascular endothelial growth factor (anti-VEGF) treatment, and myopia higher than − 6.0 dioptres were not enrolled.

Patients with intraoperative complications described in medical records such as iris prolapse, posterior capsular rupture, vitreous loss, the need for additional surgery, and the failure to use postoperative anti-inflammatory medications as prescribed were also excluded.

### Statistical analysis

Data are assumed as mean ± standard deviation (SD), except for the absolute numbers and proportions for the nominal scale. IBM SPSS Statistics 26 (SPSS Inc., Somers, NY) was used for statistical analysis. BCVA was measured as logMAR for further analysis. The conformity of numerical data to a normal distribution was evaluated using the Shapiro–Wilk. The distribution of the sample profile in nondiabetic and diabetic patients was tested using the Pearson chi-square test and the Mann–Whitney test. Non-parametric Spearman rank correlations (*rho*) were used to assess the correlations between variables, and the Wilcoxon test was used for continuous variables with a non-normal distribution.

The comparison of BCVA and CFT at the initial phase and 0 M (60 days after topical treatment) was performed using the Wilcoxon test, and throughout the treatment until 12 M, the comparison was made applying the Friedman's ANOVA test followed by Pairwise analysis with Bonferroni correction. The variation (Delta) of BCVA and CFT between nondiabetic and diabetic patients was conducted using the Mann–Whitney test.

Linear regression analysis was applied to test for significant changes in temporal trends that may have occurred during the study period, including the slope of the regression line for the change in the CFT and gain of visual acuity in logMAR over the follow-up period. The coefficient of determination (r^2^) was adjusted monthly to measure the validity of the regression model applied. p values < 0.05 were considered statistically significant.

## Results

### Baseline variables

Baseline variables regarding the patients (age and sex) were similar for nondiabetic and diabetic without DR patients. The duration of diabetes was 11.8 ± 7.2 years on average. Serum glycosylated haemoglobin (HbA1c) was available for 14 diabetics patients and this average level was 46.8 ± 12.9 mmol/mol (6.42% ± 1.12%), representing recommended glycemic control of diabetic patients. Baseline characteristics are summarized in Table 1.

**Table 1:** Characterization of the profile of diabetic and nondiabetic patients in the initial evaluation

	Group		Total	*p*
	Nondiabetic n = 21	Diabetic w/o DR n = 21		
n (%)				
Gender				
Female	11 (52.4)	13 (61.9)	24 (57.1)	0.53*
Male	10 (47.6)	8 (38.1)	18 (42.9)	
Mean ± SD				
Age (year)	67.38 ± 9.75	67.24 ± 9.92	67.31 ± 9.72	0.90**
IOP	16.48 ± 2.44	16.19 ± 2.36	16.33 ± 2.38	0.71**
BCVA (LogMAR)	0.74 ± 0.32	1.15 ± 0.35	0.95 ± 0.39	< 0.01**
CFT	537.86 ± 99.91	674.48 ± 181.49	606.17 ± 160.37	0.01**

*w/o* without, *DR* diabetic retinopathy, *n* absolute frequency, *%* relative frequency, *SD* standard desviation, *IOP* intraocular pressure, *BCVA* corrected distance visual acuity, *CFT* central foveal thickness

^*^Chi-Square, **Mann–Whitney

### Nondiabetic and diabetic patients without diabetic retinopathy with PCME: effect of DEX implant on BCVA (logMAR) improvement following initial topical treatment

The mean ± SD BCVA (logMAR) prior topical treatment in nondiabetic and diabetic patients were 0.74 ± 0.32 and 1.15 ± 0.35, respectively. Following initial topical treatment, nondiabetic and diabetic subjects presented a mean ± SD gain of − 0.11 ± 0.11 and − 0.18 ± 0.11 respectively, no difference between groups (p = 0.09) (Table 2). Subsequently, the mean gain in BCVA (logMAR) in nondiabetic and diabetic patients at month 12 after DEX implantation was − 0.35 ± 0.17 in nondiabetic subjects and − 0.55 ± 0.26 in diabetic patients, compared to 0 M (p < 0.001) (Table 3).

**Table 2:** Clinical and anatomic parameter outcomes during topical treatment

Mean ± SD	Initial	0 M	Δ (change)	p*
Total (n = 42)				
BCVA (LogMAR)	0.95 ± 0.39	0.8 ± 0.31	− 0.15 ± 0.12	< 0.001
CFT (µm)	606.17 ± 160.37	555.07 ± 149.63	− 51.1 ± 45.9	< 0.001
Nondiabetic (n = 21)				
BCVA (LogMAR)	0.74 ± 0.32	0.63 ± 0.25	− 0.11 ± 0.11	0.012
CFT (µm)	537.86 ± 99.91	494.43 ± 99.73	− 43.42 ± 53.66	0.012
Diabetic w/o DR (n = 21)				
BCVA (LogMAR)	1.15 ± 0.35	0.98 ± 0.28	− 0.18 ± 0.11	0.012
CFT (µm)	674.48 ± 181.49	615.71 ± 168.01	− 58.76 ± 36.28	0.013

*SD* standard deviation, *0* *M* 60 days after topical treatment, *BCVA* corrected distance visual acuity, *CFT* central foveal thickness, *µm* micrometer, *w/o* without, *DR* diabetic retinopathy

^*^Wilcoxon

**Table 3:** Result of the comparison of BCVA and CFT before and 12 months after the implementation of DEX treatment

	0 M	1 M	3 M	6 M	12 M	p*
Total (n = 42)						
BCVA (LogMAR)	0.80 ± 0.31	0.65 ± 0.35	0.56 ± 0.31	0.41 ± 0.22	0.35 ± 0.17	< 0.001
BCVAΔ (change)	–	− 0.15 ± 0.26	− 0.24 ± 0.26	− 0.40 ± 0.27	− 0.45 ± 0.24	
CFT (µm)	555.07 ± 149.63	361.79 ± 119.43	397.57 ± 169.18	337.48 ± 106.06	314.21 ± 77.06	< 0.001
CFTΔ (change)	–	− 193.29 ± 104.55	− 157.50 ± 126.21	− 217.60 ± 120.52	− 228.26 ± 156.79	
Nondiabetic (n = 21)						
BCVA (LogMAR)	0.63 ± 0.25	0.54 ± 0.42	0.42 ± 0.31	0.31 ± 0.25	0.28 ± 0.17	< 0.001
BCVAΔ (change)	–	− 0.09 ± 0.23	− 0.21 ± 0.26	− 0.32 ± 0.25	− 0.35 ± 0.17	
CFT (µm)	494.43 ± 99.73	318.67 ± 44.49	349.33 ± 94.13	329.43 ± 97.62	298.71 ± 42.05	< 0.001
CFTΔ (change)	–	− 175.76 ± 63.41	− 145.10 ± 125.53	− 165.00 ± 114.79	− 195.71 ± 93.23	
Diabetic w/o DR (n = 21)						
BCVA (LogMAR)	0.98 ± 0.28	0.75 ± 0.21	0.70 ± 0.24	0.50 ± 0.14	0.42 ± 0.13	< 0.001
BCVAΔ (change)	–	− 0.22 ± 0.27	− 0.27 ± 0.26	− 0.47 ± 0.28	− 0.55 ± 0.26	
CFT (µm)	615.71 ± 168.01	404.90 ± 152.84	445.81 ± 211.97	345.52 ± 115.73	329.71 ± 99.51	< 0.001
CFTΔ (change)	–	− 210.81 ± 133.20	− 169.90 ± 128.74	− 270.19 ± 103.88	− 260.81 ± 198.69	

*0* *M* 60 days after topical treatment and DEX implant occurrence, *1* *M* 1 month after DEX implant, *3* *M* 3 months after DEX implant, *6* *M* 6 months after DEX implant, *12* *M* 12 months after DEX implant, *BCVA* corrected distance visual acuity, *CFT* central foveal thickness, *µm* micrometer, *w/o* without, *DR* diabetic retinopathy

^*^Test of Friedman’s ANOVA

### Nondiabetic and diabetic patients without diabetic retinopathy with PCME: effect of DEX implant on CFT following initial topical treatment

Prior initial topical treatment, nondiabetic and diabetic patients presented with a mean ± SD CFT up to 537.86 ± 99.91 µm in nondiabetics and 674.48 ± 181.49 µm in diabetic subjects, with a mean ± SD reduction of − 43.42 ± 53.66 µm and − 58.76 ± 36.28 µm, respectively, at month 0 compared to initial (Table 2). However, there were no differences between groups (p = 0.56).

Regarding CFT fluctuations after DEX implant in nondiabetic and diabetic patients the changes were − 195.71 ± 93.23 µm (p < 0.001) and − 260.81 ± 198.69 µm, respectively, at month 12 (p < 0.001) compared to 0M (Table 3). At month 6, three eyes (all of them members of the diabetic group) presented a CFT lower than 20% compared with baseline values and were therefore treated just with one DEX implant. This resulted in a reduction in the CFT and an BCVA (logMAR) gain similar to those noted after the first injection and without any loss in the therapeutic effect.

### Impact of DEX implant and topical treatment on nondiabetic and diabetic patients with PCME

**Fig. 1:**
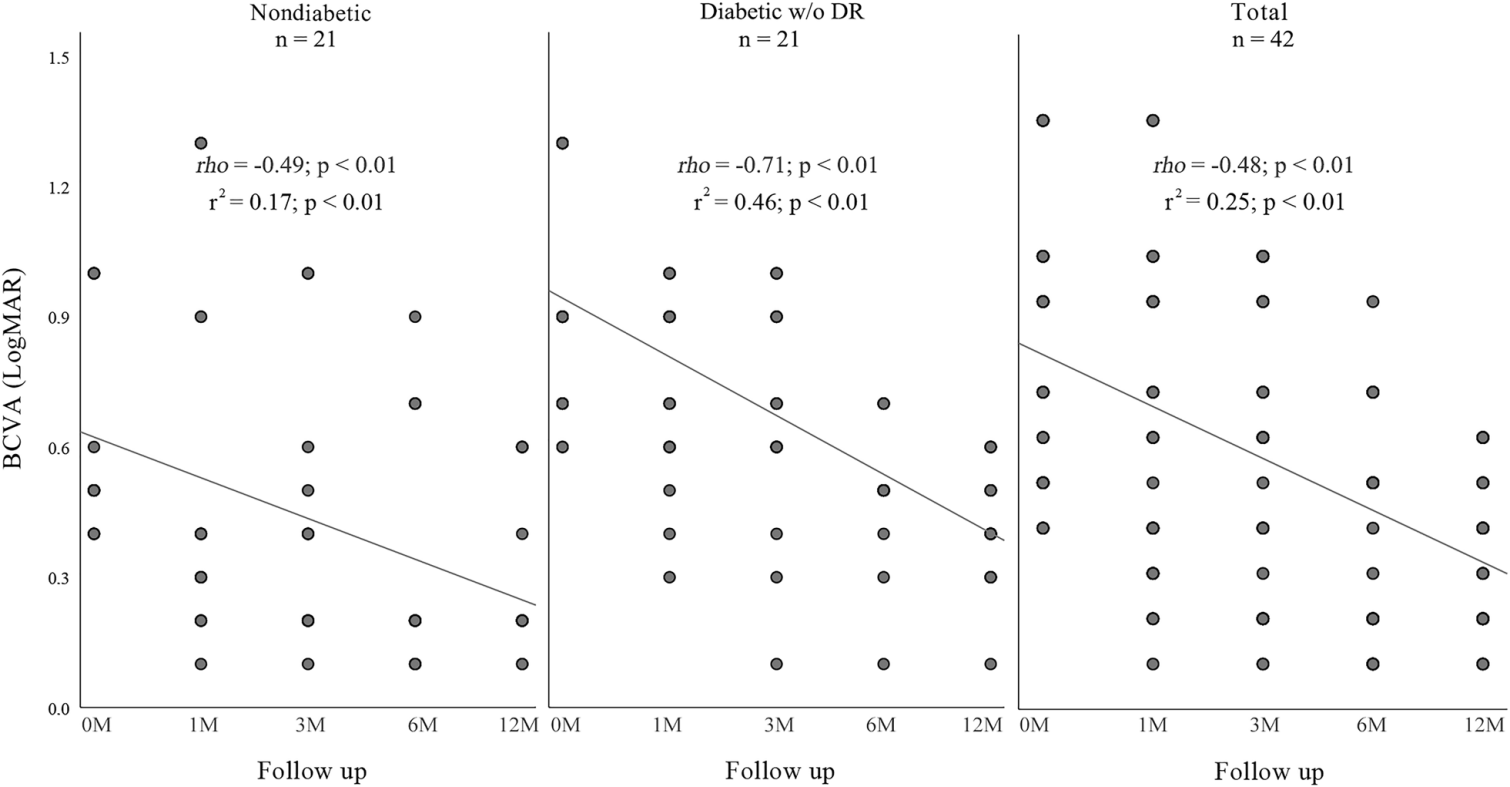
Scatter plots showing BCVA (LogMAR) and progress after intravitreal dexamethasone implant in nondiabetics (left), in diabetics without diabetic retinopathy (center) and in total of pacients (right). BCVA (LogMAR) is plotted on the y-axis and 12-month follow-up is plotted on the x-axis

Linear regression analysis demonstrated a gain in BCVA over 12 months (r^2^ = 0.25, *rho* = − 0.48, p < 0.01). Similar gain occurred in nondiabetics (r^2^ = 0.17, *rho* = − 0.49, p < 0.01). While in diabetics a strong negative correlation was found (r^2^ = 0.46, *rho* = − 0.71, p < 0.01) (Fig. 1). A permanent increase in BCVA gain was observed up to month 6 after DEX implantation. This finding was maintained until month 12.

**Fig. 2:**
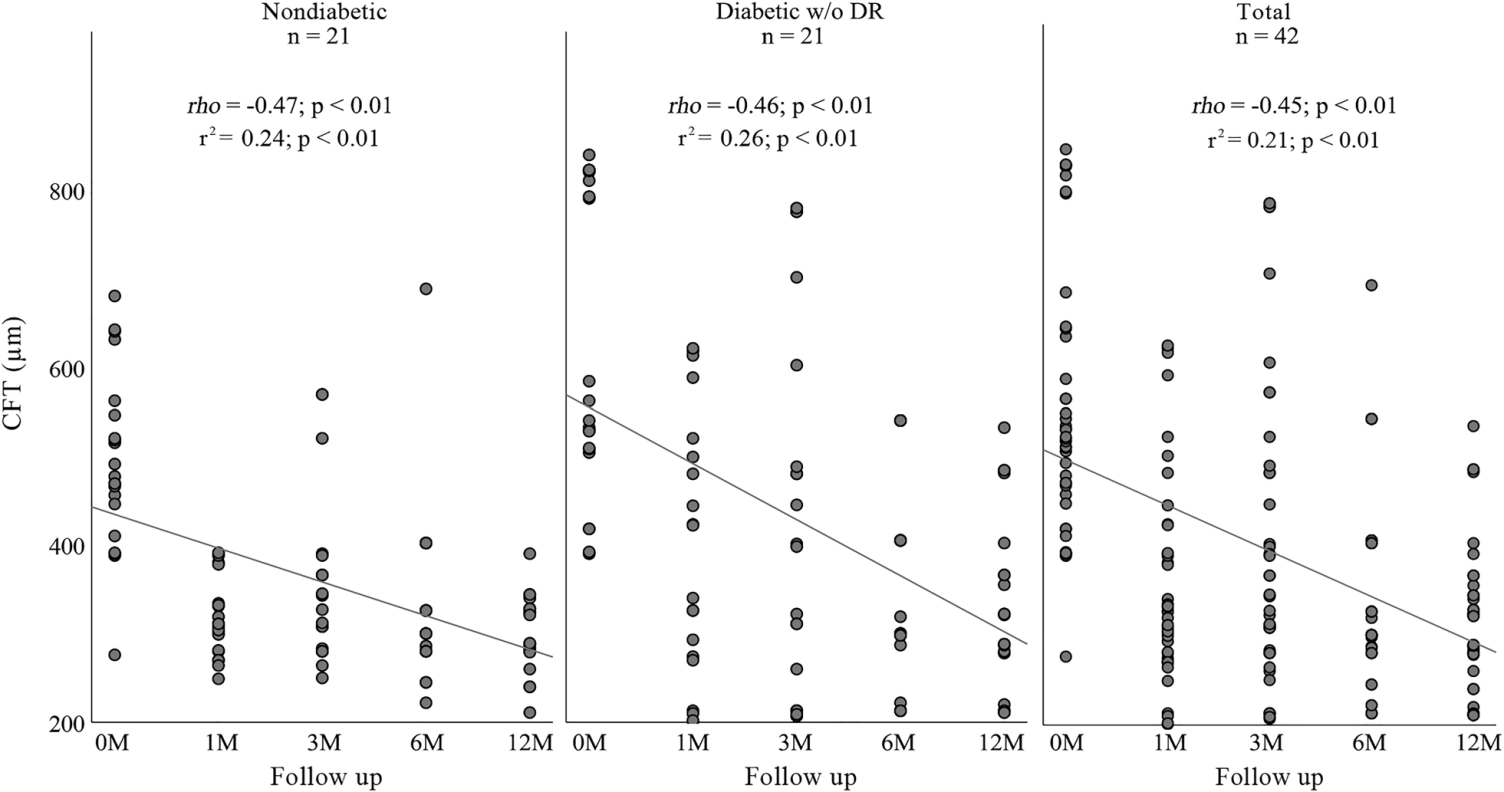
Scatter plots showing CFT(μm) and progress after intravitreal dexamethasone implant in nondiabetics (left), in diabetics without diabetic retinopathy (center) and in total of pacients (right). CFT(μm) is plotted on the y-axis and 12-month follow-up is plotted on the x-axis

There was a reduction in CFT in both groups over the 12 months (r^2^ = 0.21, *rho* = − 0.45, p < 0.01). The negative linear regression found was similar between nondiabetics (r^2^ = 0.24, *rho* = − 0.47, p < 0.01) and diabetics (r^2^ = 0.26, *rho* = − 0.46, p < 0.01) (Fig. 2). There was a significant drop in CFT at month 6, followed by a constant progression up to month 12.

**Fig. 3:**
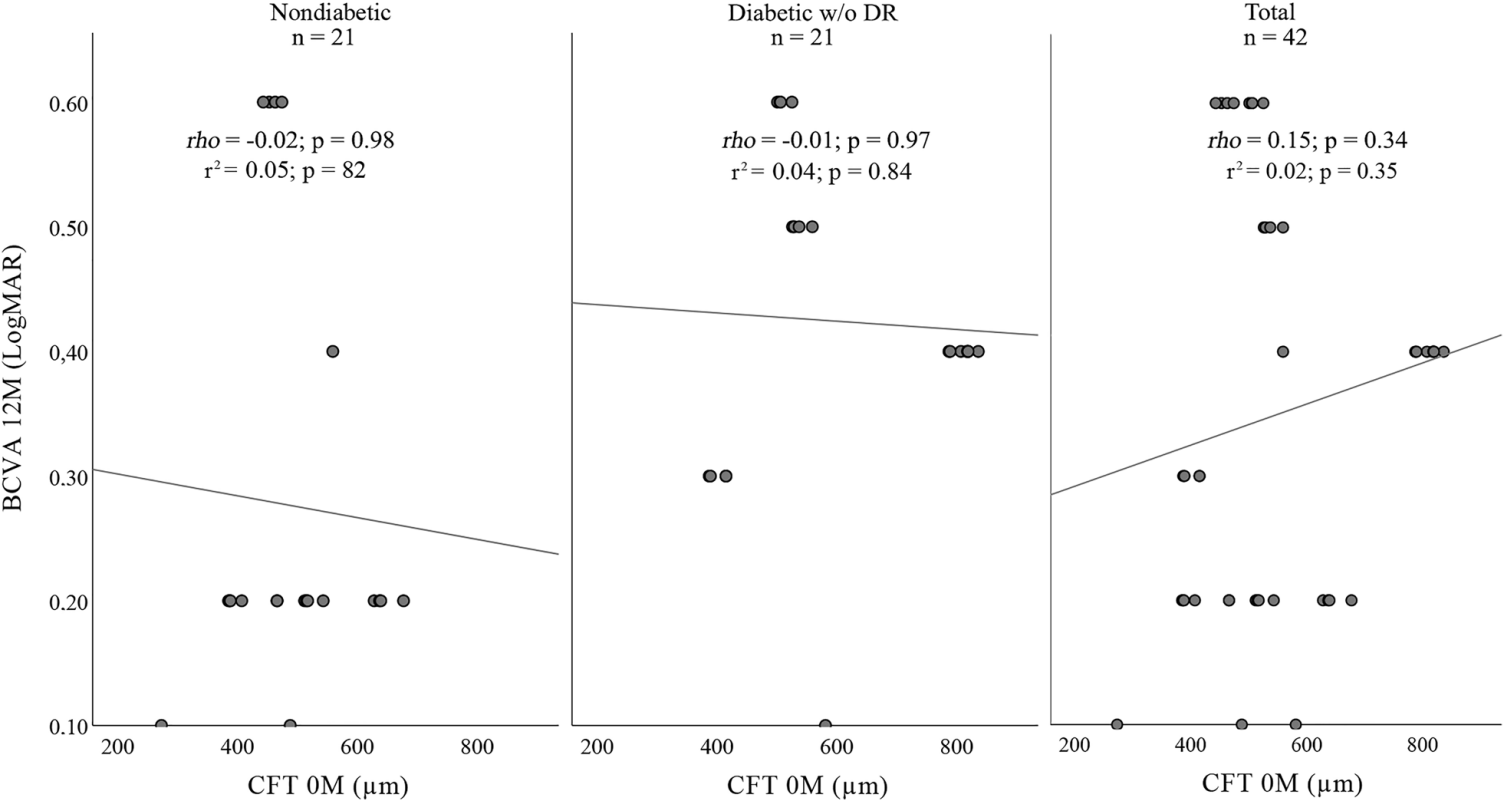
Scatter plots showing BCVA (LogMAR) after 12 months implant and CFT (μm) in the month of intravitreal dexamethasone implant (0 M) in nondiabetics (left), in diabetics without diabetic retinopathy (center) and in total of pacients (right). BCVA (LogMAR) after 12 months is plotted on the y-axis and CFT (μm) in the month of intravitreal dexamethasone implant (0 M) is plotted on the x-axis

A correlation was found between higher VA gains following topical treatment and better improvement at month 12, demonstrating that patients with PCME who responded better to topical treatment had higher BCVA improvement after DEX implantation at month 12 (Fig. 1). No correlation was found between CFT at baseline and BCVA result at month 12 (r^2^ = 0.02, *rho* = 0.15, p = 0.35) (Fig. 3).

The mean change in IOP at the end of the follow-up period was − 0.33 ± 2.54 mmHg. There was no significant difference between the mean IOP measures at initial, after the 2 months of topical therapy and at month 12 after DEX implantation. Non-parametric tests were concurrently conducted, and the results were similar.

No significant ocular or systemic side effects were observed after a single DEX implant. Furthermore, no complications, such as retinal detachment or endophthalmitis, were reported.

### Impact of a second DEX implant as retreatment in nondiabetic and diabetic patients with PCME

For 12 months, 3 diabetic patients matched the criteria for retreatment with a 2nd DEX implant. This intervention at month 6 showed a mean ± SD reduction of − 268.84 ± 138.26 µm at month 12 compared to 0M. The mean ± SD BCVA in this group of patients was − 0.22 ± 0.12, compared to 0 M. The statistical data showed the absence of bias regarding final outcomes.

## Discussion

The risk of developing PCME following a cataract surgery is influenced by individual patient factors and intraoperative events. The diabetes is a significant risk factor in the development of PCME. Our study indicates that nondiabetic and diabetic patients without DR demonstrated similar results following intravitreal DEX implant after combined topical therapy, suggesting that selected diabetic patients might have a comparable response than nondiabetic patients with PCME. Our data also suggest that patients with better anatomical and functional outcomes after initial topical treatment had greater benefits at 12 months after DEX.

It seems reasonable to hypothesize that DEX implants may represent a promising treatment option for patients who develop PCME. Inflammation and upregulated proinflammatory mediators have been identified as playing major roles in the blood–retinal barrier breakdown related to PCME [19, 31, 32]. The DEX implant regulatory approval specifies that it is indicated for the treatment of adult patients with inflammation of the posterior segment presenting as non-infectious uveitis. It could therefore be suggested that PCME represents a similar mechanism to posterior segment inflammation.

Several studies showed the benefit of DEX implantation in both refractory and naïve PCME patients [20, 26, 28–30, 33]. Dutra Medeiros et al. [18] assessed the 6-month results after a single intravitreal DEX implant in patients with recalcitrant PCME. In their study, the mean best-corrected visual acuity (BCVA) was 0.37 ± 0.26 logMAR after 6 months (p = 0.002). In a 6-month follow-up study, reported that BCVA improved by 0.21 ± 0.15 (p = 0.002), and central retinal thickness (CRT) decreased by 308 µm by month 6 (p < 0.0001) [17].

The EPISODIC 1 and 2 trials demonstrated a significant gain in early treatment diabetic retinopathy study (ETDRS) letters from baseline at month 6, which was maintained at month 12 after DEX implantation. The latter trial presents result in line with those in this study. Nevertheless, the EPISODIC group did not investigate the possible optimizing impact of combined topical treatment immediately after cataract surgery [23, 34].

The topical administration of NSAIDs, combined with steroids or not, has become common in clinical practice [34–37]. Topical nepafenac 0.1% reaches the posterior segment due to its corneal permeability characteristics seems to be superior than other NSAIDs, providing reduced risk of PCME, and it is also approved in Europe for the reduction of the risk of PCME associated with cataract surgery, including for diabetic patients [38–40]. The PREMED 1 report showed that PCME can be prevented with the combination of NSAIDs and topical steroids in nondiabetic patients, without previous treatment prior cataract surgery [41].

Although the angiographic characteristics of PCME have been reported to occur in up to 30% of asymptomatic nondiabetic patients, in diabetic patients, PCME is even more common, and up to 56% of patients with mild-to-moderate non-proliferative retinopathy might be significantly affected [8]. However, large register-based studies may fail to distinguish pre-existing DME or its progression from PCME at postoperative screening. These biases raise the concern of overestimating the impact of diabetes as a high-risk factor for PCME. Discerning between acute PCME and pre-existing DME has become possible with improved OCT techniques, with distinct patterns that might direct a targeted treatment [42–44].

The absence of DR and tight glycemic control seem to be crucial for preventing PCME in this population before cataract surgery. Our results are in agreement regarding better responses in diabetic patients compared with nondiabetic patients [35]. Elevated levels of pro-inflammatory cytokines present in the vitreous fluid of patients with diabetes may explain these better responses to the DEX implant [45, 46].

This study presents limitations that must be considered. The absence of angiographic data at month 12 should may explain the difference between nondiabetics and diabetic patients SD-OCT results. FA was performed prior to treatment with NSAIDs to identify PCME and to exclude other macular disorders, after 2 months of topical treatment, prior to DEX implantation, and after 6 months of the intravitreal implantation, as an adjuvant supporting the PCME diagnosis and the need for retreatment. HbA1c control data would provide more consistent information about the systemic conditions of the diabetic patients. The absence of a control group that did not receive topical treatment before DEX, has also to be mentioned as a limitation during recruitment of patients.

The primary endpoint was the result of DEX implantation as a treatment for PCME in nondiabetic and selected diabetic patients, aiming to establish the impact of diabetes on visual recovery and OCT improvements during a 12-month period after a 2-month course of topical combined therapy. Both groups (diabetic and nondiabetic patients without DR) showed intraretinal spaces filled with cystic fluid in the outer nuclear layer (ONL) and inner nuclear layer (INL), evidencing the presence of intraretinal fluid. At month 12, the absence of cystic spaces in the ONL and INL characterized the improvement of the intraretinal fluid.

The implantation of a second DEX implant at month 6 in 3 patients did not produce an inadvertent bias aiding them to accomplish better therapeutic results. There was no significant increase in IOP after DEX implantation, this outcome being more severe (> 30 mmHg) and significant with the use of triamcinolone compared to dexamethasone [47]. Considering the relatively small sample size, caution is needed in drawing conclusions regarding the clinically important question being investigated.

## Conclusions

Our results show that diabetic patients without DR had similar responses to DEX implants as nondiabetic patients diagnosed with PCME following uneventful cataract surgery. Subjects from both groups with better anatomical and functional outcomes after initial topical treatment also had greater benefits at 12 months after DEX. The statistical data showed the absence of bias regarding final outcomes. Late-phase studies could facilitate the evaluation of macular edema kinetics between diabetic and nondiabetic control patients.

## Data Availability

The data that support the findings of this study are available on request from the corresponding author. The data are not publicly available due to privacy or ethical restrictions.

## Abbreviations

Anti-VEGF: Antagonists of vascular endothelial growth factor
BCVA: Best-corrected visual acuity
CFT: Central foveal thickness
CRT: Central retinal thickness
DEX: Intravitreal dexamethasone
DR: Diabetic retinopathy
FA: Fluorescein angiography
HbA1c: Glycosylated hemoglobin
INL: Inner nuclear layer
IOL: Intraocular lens
IOP: Intraocular pressure
NSAIDs: Nonsteroidal anti-inflammatory drugs
ONL: Outer nuclear layer
PCME: Pseudophakic cystoid macular edema
r^2^: Coefficient of determination
rho: Spearman rank correlations
SD-OCT: Spectral domain optical coherence tomography
SD: Standard deviation
